# Design and Development of Portable Body Composition Analyzer for Children

**DOI:** 10.3390/diagnostics14232658

**Published:** 2024-11-25

**Authors:** Richa Rashmi, Snekhalatha Umapathy, Omar Alhajlah, Fadiyah Almutairi, Shabnam Mohamed Aslam

**Affiliations:** 1 Department of Biomedical Engineering, College of Engineering and Technology, SRM Institute of Science and Technology, Kattankulathur, Chennai 603203, India; rj5184@srmist.edu.in; 2 Department of Quality Assurance Cell, ANIIMS, GB Pant Hospital, Sri Vijaya Puram 744103, India; 3 Department of Applied Computer Sciences, Applied Computer Science College, King Saud University, Riyadh 11451, Saudi Arabia; oalmutiry@ksu.edu.sa; 4 Department of Information System, College of Computer and Information Sciences (CCIS), Majmaah University, Al Majmaah 11952, Saudi Arabia; fma.almutairi@mu.edu.sa; 5 Department of Information Technology, College of Computer and Information Sciences (CCIS), Majmaah University, Al Majmaah 11952, Saudi Arabia

**Keywords:** childhood obesity, body composition analyzer, bioelectrical impedance analysis, body mass index, fat free mass

## Abstract

Objectives: The aim of this study was (i) to design and develop a portable BCA device for measuring body composition parameters such as body weight, body fat (BF) %, total body water (TBW), fat-free mass (FFM), muscle mass (MM), and bone mass (BM); (ii) to validate the developed portable BCA with the Tanita MC 980 MA BCA device. Methods: For this current study, two hundred healthy and obese subjects, whose ages ranged from 8 to 12 years (8.4 ± 1.7), were considered. Results: The highest percentage difference between the two study groups was found to be in BFat (50.39%), followed by body mass index (BMI) (41.73 kg), FFM (38.32 kg), and MM (37.89 kg), and this was found to be statistically significant. The results obtained from the designed prototype of the body composition analyzer were validated using Tanita MC 980MA BCA. The overall error% was calculated as ±3% for measuring the different body composition parameters. Conclusions: Due to its low standard error and high overall accuracy, the BCA prototype demonstrates the potential to be a dependable instrument for evaluating and tracking the body composition of children.

## 1. Introduction

Obesity has emerged as a worldwide pandemic, representing an immense and growing danger to public health [1]. Around 390 million children and adolescents are vulnerable to obesity on a global scale [2]. Concerningly, the World Health Organization (WHO) estimates that there were already around 37 million children below the age 5 years who were found to be overweight in 2022 [2]. Globally, the regions most significantly impacted by childhood obesity are low- and middle-income countries [3]. By 2030, India is projected to account for around 11% of the worldwide epidemic of childhood obesity, if current patterns continue [4]. Obese children are at an increased risk of developing cardiovascular diseases such as hypertension, hyperlipidaemia, and hyperinsulinemia, which have consequences that extend beyond childhood [5,6,7].

The body composition analyser (BCA) is a device that has been carefully engineered to assess various components of the body, thereby transcending the constraints associated with traditional weight assessments [8,9,10]. This apparatus primarily serves to identify the precise proportions of water, fat, muscle, and bone within the human body [11,12,13]. BIA is a technique to evaluate the body’s resistance to harmless alternating current as it passes through water content in the body, usually between the upper and lower limbs [14]. One of the most prevalent and widely adopted approaches for measuring body composition is bioelectrical impedance analysis (BIA). The theory behind this technique is based on the natural resistance of different human body tissues to a relatively small electrical current. Adipose tissue, which comprises most of the fat-free mass, possesses a significantly greater resistance than tissues with higher water content. Due to its non-invasive nature, BIA has grown increasingly popular in the field of anthropological surveys [15]. However, the implementation of direct measurement techniques for clinical monitoring is costly. Consequently, BIA is often employed as a cost-effective alternative method.

BIA is a non-invasive method for estimating an individual’s body composition, primarily determining the proportion of fat and muscle mass. The underlying principle of BIA is based on the fact that different tissues in the human body conduct electrical currents differently [16]. BIA relies on variations in the electrical conductivity of water, muscle, fat, and bone, among other body tissues. It calculates parameters such as lean body mass, body fat percentage, and total body water by subjecting the body to low-level electrical current and measuring impedance, which is influenced by tissue resistance [17]. 

**Figure 1:**
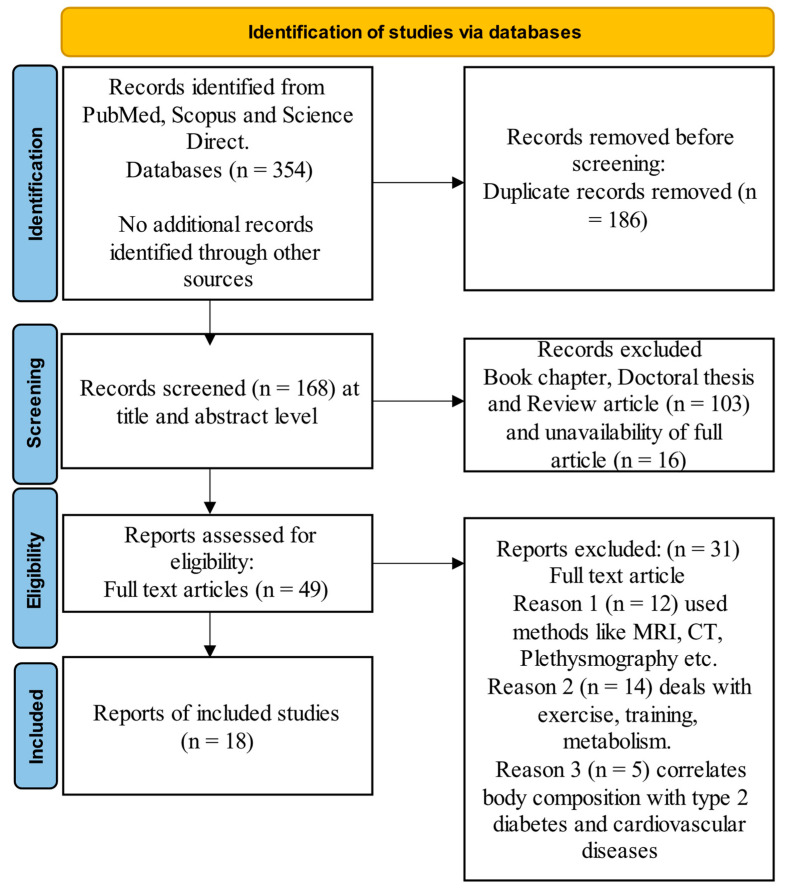
PRISMA flow chart for obesity assessment survey in children.

In this systematic literature review, we employed the Preferred Reporting Items for Systematic Reviews and Meta-Analyses (PRISMA) method to conduct a comprehensive literature survey [18] on BIA techniques for assessing body composition in children. Our rigorous search and selection process, as illustrated in the PRISMA flow diagram (Figure 1), initially identified 354 potentially relevant studies from the PubMed, Scopus, and Science Direct databases. Our search was limited to studies published between January 2000 and August 2024, focusing on English-language articles to ensure accessibility and relevance. The search’s keywords included combinations of terms such as bioelectrical impedance analysis and body composition and children and pediatric and adolescents and validation and accuracy and reliability. After a systematic screening and eligibility assessment, which included the removal of duplicates and the application of specific inclusion and exclusion criteria, we ultimately included 18 studies that met our predefined criteria for in-depth analysis.

The study by Larsen et al. (2021) evaluated the validity and reliability of the InBody 270 multi-frequency bioimpedance analysis scale for assessing body composition in children aged 10–12 years. The InBody 270 showed almost good correlations (r = 0.97–0.99) with DEXA scans for total body mass, lean body mass, and muscle mass, indicating excellent criterion validity. The authors concluded that, while the InBody 270 is a valid and reliable tool for estimating body composition in this age group, caution is needed when interpreting muscle mass values due to these biases [19].

Wells et al. (2021) utilized BIA to assess the physical development and body composition of schoolchildren aged 7–16 years, revealing significant sex-specific differences in body composition parameters. The researchers identified an inverse correlation between relative SMM and fat mass (FM), suggesting that SMM contributes to obesity prevention in schoolchildren [20]. Divala et al. (2022) developed and validated bioelectrical impedance-based prediction equations for estimating body composition in Malawian adolescents aged 10–18 years, using a cross-sectional design with 186 participants. The researchers created novel equations for estimating fat-free mass (FFM) and TBW based on resistance index, sex, age, and weight, which showed good agreement with the reference deuterium oxide dilution method [21]. Van et al. (2021) evaluated the reproducibility and established reference values for body composition measurements in children and adolescents aged 3–18 years using the Body Composition Monitor (BCM) device. The study provided gender-specific smoothed percentile curves and reference values for parameters such as fat mass, lean tissue mass, extracellular water, and total body water, offering valuable benchmarks for clinical and research applications. These findings support the BCM as a reliable tool for monitoring body composition in paediatric populations, with implications for addressing paediatric obesity, nutrition, and chronic disease assessment [22].

Maw et al. (2024) developed a BIA-based prediction equation for estimating body composition in rural children aged 4–8 years in Myanmar, with the equation showing a high R^2^ of 0.891 (*p* < 0.0001). The prediction equation for total body water demonstrated a small bias of 0.01 kg (0.1%) and limits of agreement (LOA) of ±1.0 kg (9.8%). Significant differences were observed in BIA variables between validation and cross-validation groups, with girls showing higher impedance and resistance than boys, resulting in a lower resistance index [23].

Van Zyl et al. (2019) developed a new BIA-based prediction equation for estimating FFM in black, pre-adolescent South African children, achieving an adjusted R^2^ of 0.9544, indicating normally distributed residuals (*p* > 0.05). The equation obtained showed improved agreement with FFM measured by DXA, with a mean difference of 0.5 ± 4.9 kg and 95% limits of agreement between −9.2 and 10.2 kg. Cross-validation repeated five times confirmed the consistency of the regression equations, with all coefficients significant (*p* < 0.01) [24].

Despite the significant advancements in BIA for body composition assessment in children, our literature review revealed a notable gap in recent research (2017–2024) specifically focused on portable BIA devices for pediatric populations. While studies have validated BIA methods in children and explored their use in various clinical settings, there is a lack of comprehensive research on the design, development, and validation of portable BCA prototypes tailored for children aged 8–12 years, particularly those addressing both normal weight and obese populations. Our study aims to fill this gap by developing and validating a novel portable body composition analyser prototype specifically designed for children. By incorporating advanced technologies such as the AD5933 IC chip, photosensor, and ATMEGA328 microcontroller, and validating these against established methods like the Tanita MC 980 MA BCA, our research contributes to the field by providing a reliable, accessible, and child-specific tool for body composition analysis. Thus, there is a need for age-appropriate, portable devices that can accurately assess body composition in diverse pediatric populations, potentially improving health monitoring and interventions in children.

The aim of this study was (i) to design and develop a portable body composition analyser for children and to measure parameters such as body mass index (BMI), body fat (BF) %, total body water (TBW), fat-free mass (FFM), muscle mass (MM), and bone mass (BM) from the developed prototype, and (ii) to predict a regression equation for each body composition’s parameters using the developed portable BCA, and to validate it with Tanita MC 980 MA BCA.

The paper is organized as follows: Section 1 is an introduction to the BCA device and its related survey. Section 2 focuses on the detailed methodology, illustrating the workflow and components of the developed BCA prototype. Section 3 illustrates the results, explaining the BCA operating procedure, the developed regression equations, and validity of the device. Section 4 deals with a discussion of the results with respect to the related literature, and Section 5 indicated the conclusion and future work of the proposed work.

## 2. Materials and Methods

### 2.1. Participants

This study was conducted on two hundred (N = 200) healthy normal and obese subjects, with ages ranging from 8 to 12 years (8.4 ± 1.7), out of which one hundred (n = 100) were boys and one hundred (n = 100) were girls. The study camp was conducted in the Sivananda Gurukulam School, Kattankulatur, Tamil Nadu, India, during the month of September 2019. It was requested that a detailed questionnaire regarding the health assessment of the child be filled out by the parents. Signed informed consent was obtained from all the parents after explaining the purpose of the study. The institutional (SRM Medical College, Hospital and Research Center, Kattankulatur, Tamil Nadu, India) ethical committee approved this current study and provided an ethical clearance number of 1740/IEC/2019.

Children between the ages of 8 and 12 years are included in the research. Children were included depending on their weight status, such as being classified as normal or obese, based on BMI percentiles or z-scores for their age and gender. The Centers for Disease Control and Prevention (CDC) divided the children into underweight, normal, over-weight, and obese according to the BMI for age weight status [25]. The different categories of children with their percentile is given in Table 1. For this current study, underweight participants were excluded, and children above the 95th percentile were considered as the obese group. Furthermore, children with certain medical issues that impact body weight, such as genetic abnormalities, hormonal disorders, or chronic diseases, were excluded from participating. Lastly, children older than 12 years were excluded from this study.

Specifically, participants were advised to maintain a stable hydration status by avoiding large fluid intakes for at least two hours prior to measurements. All measurements were conducted within a consistent time window each day between 9:00 a.m. and 12:00 p.m. to account for diurnal variations in body composition. Additionally, participants refrained from vigorous physical activity for at least 24 h before measurements, as such activities could impact hydration and muscle composition. These steps were taken to ensure that variability in the body composition measurements was minimized, thereby enhancing the accuracy and reliability of the results obtained using the developed portable BCA device.

### 2.2. Overall Workflow of Proposed BCA

The study involved 200 individuals in total and these individuals were categorized into two distinct study groups. Then, the various body composition measurements were conducted using the proposed BCA for children. The parameters which were evaluated using the proposed BCA were BMI, BF%, TBW, FFM, MM, and BM.

The proposed portable BCA can serve as an instrument employed to acquire these kinds of body composition measurements. The information gathered from these assessments could be analysed to gain a deeper understanding of the body composition parameters. Furthermore, regression equations for each body composition parameter were developed. In addition, the accuracy of the estimated body composition parameters were derived from the proposed portable BCA and validated by contrasting them with the outcomes derived from the widely employed Tanita MC 980 MA BCA.

**Figure 2:**
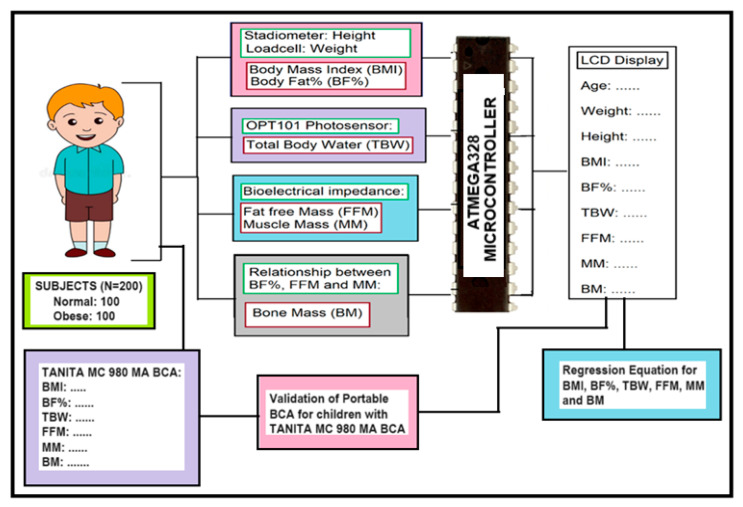
The block diagram of the proposed body composition analyzer for children in detection of child obesity.

The proposed portable BCA consists of a load cell, height scale, AD5933 IC chip with electrodes, ATMEGA328 microcontroller, OPT101 photo sensor, and LCD display. The block diagram of the proposed BCA is illustrated in Figure 2. The BMI was measured by calculating the height and weight of the children using the physical height scale and load cell, respectively. The photosensor (OPT101) uses near infrared interactance to measure total body water through finger bed region. The peak absorption for water is 970 nm. AD5933 IC uses the BIA principle and helps in measuring FFM and MM. BF% is defined as the proportion of fat mass relative to total body mass, expressed as a percentage. This measure reflects the body’s fat composition, distinguishing it from other components such as muscle, bone, and water. This addition clarifies the significance of BF% as a parameter in body composition analysis, particularly for evaluating body composition in children. Lastly, bone mass was calculated using the relationship between BF%, FFM, and muscle mass. Then, all these raw data were fed into the ATMEGA 328 microcontroller, into which the program was fed, to analyse the incoming raw data and convert them into the actual values of each parameter. Finally, these parameters were displayed on the LCD display so that the data could be analysed and collected for further evaluation.

### 2.3. Components of Proposed BCA Prototype

#### 2.3.1. Loadcell and HX711 IC

**Figure 3:**
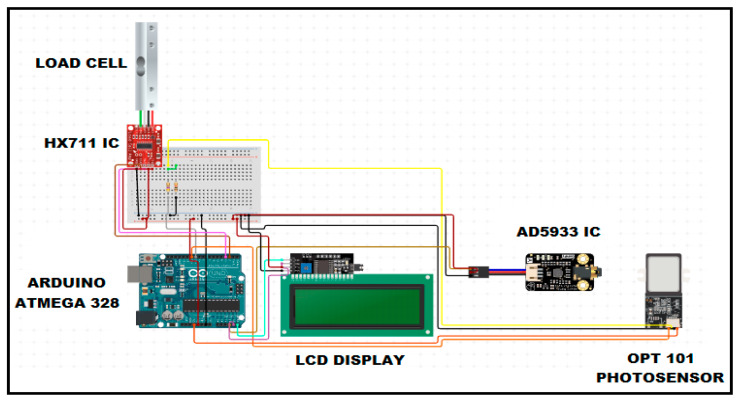
Circuit diagram of main components of proposed portable BCA.

The load cell serves as a sensor that transforms various physical quantities, including strain, pressure, weight, and force, into corresponding electrical signals. Consequently, the electrical output of the load cell varies directly with the magnitude of the applied load. In this current prototype, the 60-kg aluminium alloy load cell is equipped with an associated HX711 amplifier module, as depicted in Figure 3. The HX711 is a 24-bit analog-to-digital converter (ADC) which amplifies an analog signal from the load cell and outputs a reliable and precise digital signal. The load cell itself weighs 0.31 kg and features four leads, which are connected to a power source ranging from 5 V to 10 V. The load cell derives its power from the HX711 chip which eliminates the necessity for an additional power source. In this instance, we utilized a durable 2-inch wooden board for the framework to make it easier for the children to stand upon, along with a wooden board serving as the base to make the whole platform steady and secure for the subjects.

When a load is applied to the load cell (X_1_ = 0 kg), the ADC value is derived from the HX711 IC, yielding Y_1_. Subsequently, when a child stands on the platform, exerting a strain on the load cell (X_2_ = 30 kg), the ADC value is recorded as Y_2_. Following this, the calibration factor m as per Equation (1) is calculated.

(1) $$m=\frac{{(X}_{2\;}-X_{1})}{{(Y}_{2\;}-Y_{1})}$$

It is important to note that X_1_ consistently represents the absence of load, serving as an offset, C. Hence to achieve a calibrated measurement, the ADC value from the HX711 IC is utilized which is then converted into weight measurements, per Equation (2) [26].

(2) $$Weight\left(Kg\right)=\left(ADC\;value*m\right)-C$$

#### 2.3.2. OPT101 Photosensor

The photoelectric detector OPT101 represents an integrated photodiode equipped with an on-chip transimpedance amplifier. The integration of a photodiode with a transimpedance amplifier on a single chip design can effectively minimize current leakage, reduce noise, and mitigate stray capacitance-induced gain peaks [27]. In terms of circuitry design, the OPT101 features a bandwidth of 2.5 kHz at an external 5-MΩ feedback resistor and an external 10-pF capacitor [28]. The DC gain of the OPT101 is 6 × 106 V/A, and the actual working voltage is 5 V [29].

**Figure 4:**
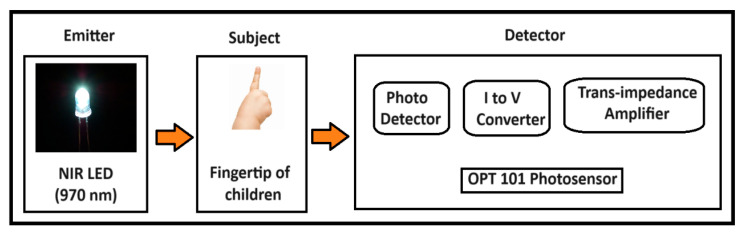
Functioning of OPT 101 photosensor.

The peak absorption for water occurs at 970 nm [30], and the developed prototype employs a single LED that emits light at a 970-nm wavelength. The initial step in the process is the identification of the light source as it is imperative that the light incident on the subject remains intact with the fingertip. The transmitted light can penetrate through 0.4 to 0.8 inches of dermis layer, necessitating that the detector can respond to this light for the absorption of near-infrared light by body water. Below the fingertip, the detector is composed of the OPT101 sensor, as depicted in Figure 4. This sensor is an optoelectronic device integrated with current-to-voltage converters and operational amplifiers which are packed within a single IC. The OPT101 sensor demonstrates high sensitivity towards the 970-nm wavelength coupled with additional advantages such as a small size, low cost, compact design, high responsivity of 0.57 V/µW, reduced leakage error, and suitability for use in portable devices [31].

Specifically, the light intensity (L) signal detected by the OPT101 photosensor is derived from the propagation of light through the assessed tissue. This light intensity is subsequently converted into optical density (OD) through the implementation of Equation (3).

(3) $$OD=-{log}_{10}\frac{L}{L_{0}}=-\log_{10}\frac{V}{V_{0}}$$

where, L_0_ represents the initial light intensity. The terms V_0_ and V denote the respective initial converted voltage signals in response to L_0_, and the detected light intensity converted into voltage. For example, if V is obtained as 0.05 V and the V_0_ was registered as 0.5 V, then OD = ${-log}_{10}0.05/0.5$, which is ${-log}_{10}(0.1)$, and we obtain OD as 1. Once the value of OD is derived, then the transmission factor (T) is computed using Equation (4) [32].

(4) $$T=10^{-OD}$$

Through Equation (4), we derive the T value as 0.1 (T $=10^{-1})$, then the verification of V and V_0_ is carried out using Equation (5) [33].

(5) $$V=V_{0}*T,$$

Here, the V value is verified using Equation (5) and we derive V as 0.05 V (V = 0.5 * 0.1). Finally, the optical current (I_OC_) is computed using Equation (6).

(6) $$I_{OC}=Responsivity*V$$

where, responsivity for 970 nm wavelength LED is computed as 0.42 A/W, which was derived from the datasheet of the OPT 101 photosensor. Using Equation (6), I_OC_ = (0.42 A/W * 0.05 W), we obtain the optical current as 21 mA.

#### 2.3.3. AD5933IC

AD5933 is a highly accurate impedance converter system that integrates a built-in frequency generator with a 12-bit, 1 MSPS, and analog-to-digital converter (ADC) [34]. This frequency generator enables the excitation of an external complex impedance using a known frequency. The resulting impedance signal is then sampled by the ADC. For impedance estimation, the AD5933 applies the DFT over the sampled current signal and the reference sinusoidal signal, performing a 1024-point DFT for a 100-kHz frequency sweep to obtain the real and imaginary components of the impedance [34].

(7) $$\frac{1}{Z(w)}=\frac{V_{out\;}\left(w\right)-V_{in}(w)}{I_{load\;}(w)}$$

where Z(w) represents the impedance being measured, V(out) is the measured frequency, V(in) is the voltage at the input of the sensing stage, and I(load) is the current flowing through the impedance being measured.

#### 2.3.4. Arduino ATmega328

The functioning of the Arduino ATmega328 microcontroller controls the input and output procedures and processing code, and interfaces with numerous sensors and devices. The ATmega328 performs programming from its flash memory. It offers enough digital and analog I/O pins to interface with various sensors and components used in body composition analysers, such as electrodes, ADCs (e.g., AD5933), display units, and communication modules [35]. It is capable of reading sensor data, making decisions based on those data, and operating via controlling output devices.

#### 2.3.5. Liquid Crystal Display (LCD)

A 16 × 2 LCD is selected for this portable prototype and provides two lines of 16 characters each, which is sufficient to display key information such as weight, age, height, FFM, BMI, MM, TBW, BF%, and some crucial instructions like to stand on the weighing platform or to place a finger on the OPT sensor. The 16 × 2 LCD also offers good visibility with a backlit screen, making it easy to read in various lighting conditions.

### 2.4. Experimental Setup

The temperature in the study room was kept at 21 °C, and children were advised to dress comfortably in light cotton clothing. Firstly, the participants’ names, ages, genders, and heights were recorded. Then, the three recording surface electrodes were attached to the subjects’ hands. Then, the device was switched on to feed the age and height of the subject. After feeding the related details, the subject was asked to stand on the device in bare feet for 15 s to record their weight. Then, the subject was asked to place their finger on the photosensor of the device for 10 s. After that, the device automatically calculated all the parameters, and the output values were displayed on the LCD screen. The parameters such as age, height, weight, BMI, FFM, BF %, TBW, MM, and BM were displayed on the LCD Screen.

### 2.5. Estimation of Various BCA Parameters

In this study, we employed the HX711 IC with a 60-kg load cell, which is appropriate for the weight measurement of children. Once the age and height were registered into the proposed BCA, Equation (2) was used to derive the weight of the subject standing on the load cell attached to the platform. Finally, as the weight (W) and height (H) of the subject were determined and registered by the microcontroller, the BMI and BF% could be calculated using Equations (8) and (9), respectively [36].

(8) $$BMI=\frac{W\;\left(kg\right)}{H\;\left(m\right)2}$$

(9) $$BF\%=\left(1.15*BMI\right)-\left(0.7*R\right)-\left(3.6*S\right)+1.4$$

where, R denotes age and S” represents gender, coded as 0 for girls and 1 for boys, to account for gender-based physiological differences in body composition. This coding allows for appropriate adjustments when calculating body composition parameters, as gender can influence factors such as body fat percentage and muscle mass. In this proposed BCA prototype, we employed a bipolar surface electrode system attached to the hand and foot of the subject. The AD5933 IC is set up to produce a sinusoidal excitation signal. This excitation signal’s output is linked to electrodes 1 and 2. It is important to control and keep the current’s magnitude at 0.25 mA to prevent any adverse effects on the heath of the children. Thus, an alternating current (f = 50 kHz) was chosen to assess the impedance effects within the cells. The drop in voltage across the body was measured at electrode 2. This measurement was returned to the AD5933 via an analog front-end circuit to refine the signal. In the end, AD5933 determined the impedance (Z) by evaluating the phase and magnitude of the voltage in relation to the current. Once the impedance value was obtained, the FFM and MM could be determined using Equations (10) and (11), respectively.

(10) FFM(kg)=(0.742×H(cm)2/impedance(Ω))+(0.151×W(kg))+1.613

(11) MM=[(H)2/Resistance∗0.401)+(S+3.825)+(R∗(−0.071)]+5.102

Then, the child was asked to place their fingertips on the OPT 101 photosensors, as shown is Figure 4. Here, the optical density was calculated using Equation (3). Once the OD value was known, then the output voltage was verified using Equation (5) to obtain a highly precise calculation of optical current (OC), which was determined using Equation (6). This optical current value was registered by the microcontroller, and then processed further to estimate the amount of TBW in children, using Equation (12) [37]. Lastly, BM was calculated by utilizing the subject’s weight, height, and MM values already recorded in the microcontroller through Equation (13). Finally, all the study body composition parameters, namely, BMI, BF%, TBW, FFM, BM, and MM were displayed on the LCD screen of the proposed BCA prototype, through which data could be collected for further processing.

(12) TBW=(0.698∗R)+(0.414∗H)–(0.491∗W)+(2.638 x log (1/ OC))+14.61

(13) BM=[0.328 (W)+0.339 (H)−29.533]−MM

### 2.6. Performance Metrics

The performance metrics such as accuracy, mean accuracy, and mean average error can be calculated using the following formulas:

(14) $$Accuracy=\left(1-\frac{\left|PBCA-TBCA\right|}{TBCA}\right)*100\%$$

(15) $$\mathrm{Mean}\mathrm{Accuracy}=100-\frac{\sum \left|\;Obese\;Diff\;\%\;\right|+\sum \left|Normal\;Diff\;\%\;\right|}{2*Number\;of\;Parameters}$$

(16) $$\mathrm{Mean}\mathrm{Avg}\mathrm{Error}=\frac{\sum \left|\;Obes\;TBCA-Obes\;PBCA\right|+\sum \left|Norm\;TBCA-Norm\;PBCA\;\right|}{2*Number\;of\;Parameters}$$

### 2.7. Statistical Analysis

The statistical software SPSS (19.0) was utilized to perform regression analysis, ANOVA, reliability, and student’s *t*-test. An analysis of variance (ANOVA) is a statistical technique utilized to compare the means of multiple groups. The student’s *t*-test compares two groups’ means to find significant differences. This study compares prototype body composition analyser measurements to benchmark device Tanita MC 980 MA BCA measurements. Regression equations, mathematical models of independent–dependent connections, are fundamental to this study. Independent factors are considered from BCA data, whereas dependent variables include BMI, BF%, TBW, FFM, MM, and BM.

We used the Shapiro–Wilk test to assess the normality of our data distribution. The Shapiro–Wilk test is particularly useful for small-to-moderately-sized sample sets, as it evaluates whether the data follow a normal (Gaussian) distribution, which is essential for the validity of parametric statistical tests like ANOVA and regression analysis. In our study, the Shapiro–Wilk test was applied to all primary body composition parameters (e.g., BMI, body fat percentage, fat-free mass, total body water, muscle mass, and bone mass) for both normal and obese groups. This allowed us to confirm that the data met the assumption of normality, ensuring that the statistical methods used were appropriate and that the results were reliable.

## 3. Results

### 3.1. BCA Operation Procedure

**Figure 5:**
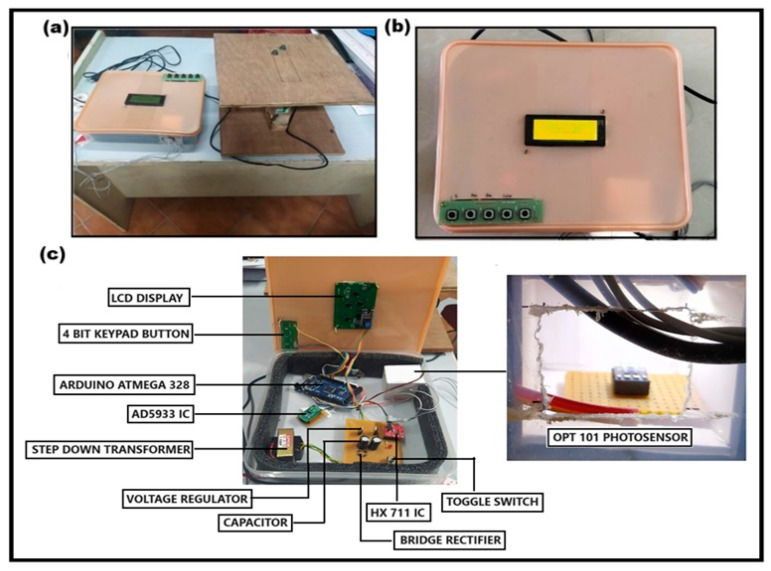
Body composition analyzer (BCA) prototype and internal component layout. (**a**) External setup of the BCA prototype, showing the weighing platform on the left and the main control unit housing the display and keypad on the right; (**b**) close-up view of the control unit interface, with a 16 × 2 LCD display for measurement outputs and a four-button keypad for user inputs like age and height; and (**c**) internal component layout of the proposed BCA device.

In the proposed BCA prototype, once both age and height are entered, the clock starts for 10 s within which the child is asked to stand on the wooden apparatus attached to the load cell. Then, children are asked to place finger on the sensor for 10 s. Once all these procedures are completed, then all the BC parameters are displayed on the LCD screen. Firstly, age, height, BF%, BMI, and FFM are flashed on the LCD screen for 15 s, followed by flashing age, weight, TBW, MM, and BM of the same subject for 15 s. The same procedure was repeated for all 200 (100 normal and 100 obese) study subjects, and their BC data were collected for further analysis. The outlook of the proposed BCA device is illustrated in Figure 5.

### 3.2. Demographic Variables

The descriptive statistics of obese and normal subjects for the total population (N = 200), boys (n = 100), and girls (n = 100) categories are illustrated in Table 2. The mean age of the obese subjects in the total population was found to be 8.4 ± 0.5 years, whereas, as the normal subjects’ mean age was 8 ± 0.01 years.

Table 2 shows that, for the total population studied, the highest percentage difference was found in BMI (41.73%) followed by BF% (37.51%), and the least percentage difference was found in FFM (20.21%) among the six body composition parameters. The mean weight of the obese participants in the total population was calculated as 43.5 ± 0.70, whereas, 29.51 ± 3.53 was obtained for the normal participants in the total population and it was found to be statistically significant. However, the parameter height was found to have the least %difference of 1.05%, and was also found to be not significant for the total population. In the parameter TBW, the percentage difference of 30.4% was obtained for the total population studied.

**Figure 6:**
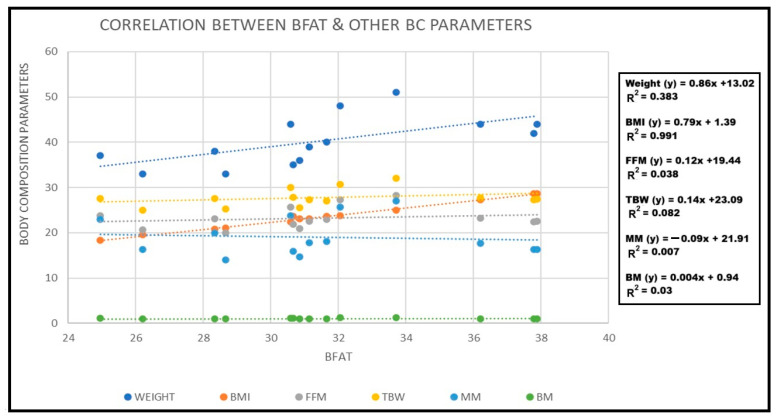
Correlation between BF% and remaining body composition parameters, namely, weight, BMI, FFM, TBW, MM, and BM.

Table 3 represents the Pearson correlation matrix for obese children (n = 100). The BF% was positively associated with all the parameters, namely, weight (r = 0.43), BMI (0.82), TBW (0.31), FFM (0.43), MM (0.44), and BM (0.38). Hence, as BF% increases, weight, BMI, TBW, FFM, MM, and BM increase in the body. Likewise, if BMI increases in the human body, then weight (r = 0.241, *p* < 0.05), TWB (r = 0.296, *p* < 0.05), BF% (r = 0.827, *p* < 0.01), MM (r = 0.175, *p* < 0.05), and BM (r = 0.201, *p* < 0.05) increase, but FFM (r = −0.047 *p* < 0.05) decreases in the human body. The MM depicts a significant negative correlation with only TBW (r = −0.002) and is also not significant (*p* > 0.05), whereas, the BM value only positively correlated with BMI (r = 0.201; *p* < 0.05), FFM (r = 0.166, *p* < 0.005), and BF% (r = 0.382; *p* < 0.05). Figure 6 shows the correlation of the measured BF% with the measured body composition parameters, namely, weight, BMI, FFM, TBW, MM, and BM.

### 3.3. Regression Model and Validation

Table 4 depicts a stepwise linear regression model which was constructed by considering BMI as a dependent variable and BF%, FFM, TBW, MM, and BM as independent variables.

The obtained optimum regression equations used to estimate the BMI level significantly using the available measured variables is given in Equation (17).

(17) BMI=0.417 (A1)+0.358 (A2)−0.090 (A3)−0.139 (A4)+7.654 (A5)−0.833

where, A_1_, A_2_, A_3_, A_4_, and A_5_ represent BFAT, FFM, TBW, MM, and BM, respectively.

A regression model was built containing BF% as a dependent variable and FFM, TBW, MM, BM, and BMI as independent variables. The acquired optimum regression equation to estimate BF% is given in Equation (18).

(18) BF%=0.560 (B1)+0.450 (B2)−0.116 (B3)−0.390 (B4)−0.477 (B5)−3.081

where, B_1_, B_2_, B_3_, B_4_, and B_5_ represent FFM, TBW, MM, BM, and BMI, respectively.

A regression model was built containing FFM as a dependent variable and TBW, MM, BM, BMI, and BF% as independent variables. The acquired optimum regression equation to estimate FFM is given in Equation (19).

(19) FFM=0.144 (C1)+0.413 (C2)+3.586 (C3)+0.096 (C4)+0.310 (C5)+1.166

where, C_1_, C_2_, C_3_, C_4_, and C_5_ represent TBW, MM, BM, BMI, and BFAT, respectively.

A regression model was built containing TBW as a dependent variable and MM, BM, BMI, BFAT, and FFM as independent variables. The acquired optimum regression equation to estimate BFAT is given in Equation (20).

(20) TBW=0.131 (D1)+6.467 (D2)– 0.024 (D3)+0.103 (D4)+0.142(D5)+12.052

where, D_1_, D_2_, D_3_, D_4_, and D_5_ represent MM, BM, BMI, BFAT, and FFM, respectively.

A regression model was built containing MM as a dependent variable and BM, BMI, BFAT, FFM, and TBW as independent variables. The acquired optimum regression equation to estimate BFAT is given in Equation (21).

(21) MM=−1.502 (E1)−0.095 (E2)−0.069 (E3)+1.061 (E4)+0.342 (E5)−9.155

where, E_1_, E_2_, E_3_, E_4_, and E_5_ represent BM, BMI, BFAT, FFM, and TBW, respectively.

A regression model was built containing BM as a dependent variable and BMI, BFAT, FFM, TBW, and MM as independent variables. The acquired optimum regression equation to estimate BFAT is given in Equation (22).

(22) BM=0.009 (F1)+0.015 (F3)+0.027 (F4)−0.002 (F5)−0.194

where, F_1_, F_3_, F_4_, and F_5_ represent BMI, FFM, TBW, and MM, respectively.

The data obtained from BCA are then validated using the Tanita MC 980 MA BCA. Each parameter of the BCA is compared and evaluated with Tanita MC 980 MA BCA for the two groups, namely, obese and normal. Based on the observations, the overall error% was calculated as ± 3% for measuring the different body composition parameters.

The Table 5 metric shows the average difference between measurements taken by different raters with a 95% confidence interval. The weight (PBCA) has a mean difference of 3.49 (2.96–4.01), indicating a small but consistent difference between raters. In contrast, BFat (PBCA) shows a mean difference of 0.07 (−0.06–0.12), suggesting almost perfect agreement. Standard Error of Measurement (SEM) values are low across all measurements such as 0.01 for BM (PBCA) and 0.45 for FFM (PBCA), indicating high precision in the measurements. The Mean Difference (MD) values are small such as 0.13 for BFat (PBCA) and 0.58 for MM (PBCA), further supporting the reliability of the measurements. The relative reliability (95% CI) expressed as the Intraclass Correlation Coefficient (ICC) indicates the consistency of measurements. High values such as 1.78 (0.78–2.78) for weight (PBCA) and 0.31 (−0.69–1.31) for BFat (PBCA) suggest excellent reliability. The inter-rater reliability (95% CI) metric also shows high values such as 0.95 (0.93–0.96) for FFM (PBCA) and 0.96 (0.93–0.96) for BFat (PBCA), indicating strong agreement between raters. Overall, Table 5 demonstrates that both PBCA and TBCA devices provide reliable and consistent measurements across various parameters. The small mean differences and high reliability coefficients suggest that the devices are effective at providing accurate body composition measurements with minor variations in agreement levels.

### 3.4. Bland–Altmon Plots

**Figure 7:**
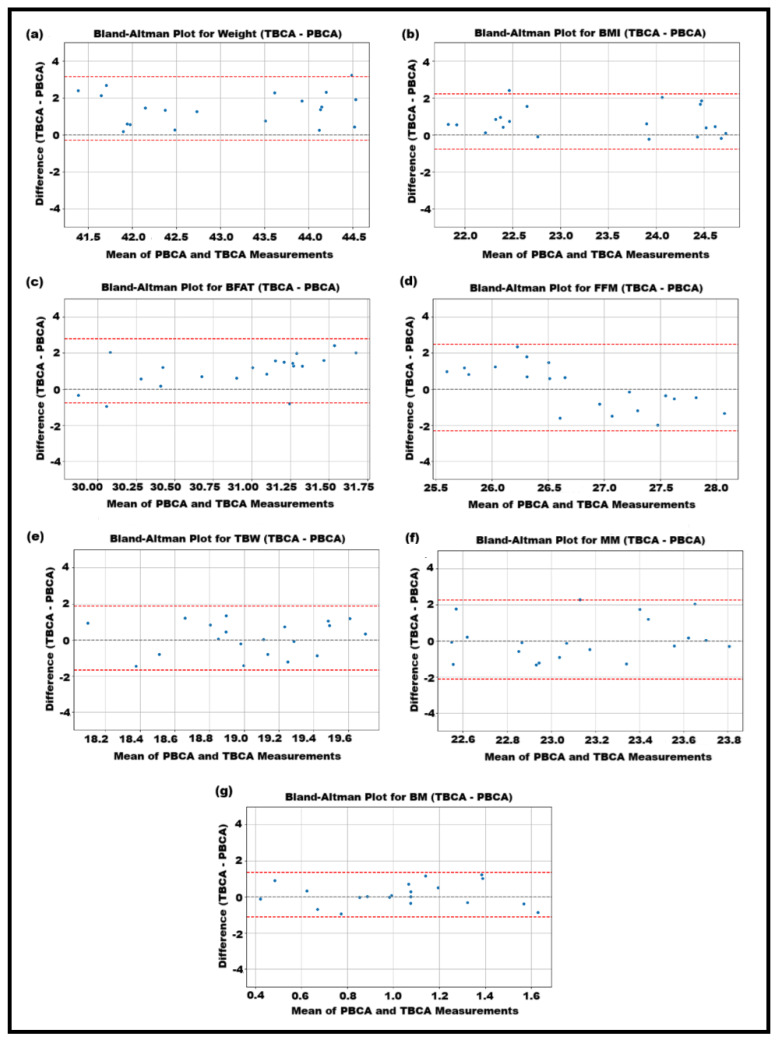
Bland–Altman plots comparing TBCA and PBCA measurements for seven body composition parameters: (**a**) weight, (**b**) BMI, (**c**) BFat%, (**d**) FFM, (**e**) TBW, (**f**) MM, and (**g**) BM. The central dotted line represents the mean difference (bias) between the two methods, indicating whether one method tends to overestimate or underestimate compared to the other. The upper and lower dotted lines represent the limits of agreement (mean difference ± 1.96 × SD), within which 95% of the differences are expected to lie. Points falling outside these limits indicate potential outliers or disagreement between the methods.

The Bland–Altman plots presented in Figure 7 reveal that TBCA and PBCA generally agree well on Weight and BMI measurements, with small biases of approximately 0.5 kg and 0.2 kg/m^2^, respectively, and narrow limits of agreement (±2 kg for Weight and ±1 kg/m^2^ for BMI). However, for Body Fat percentage, TBCA underestimates by about 2% compared to PBCA, with wider limits of agreement (±5%), indicating more variability. Fat-Free Mass shows the largest discrepancy, with TBCA overestimating by about 3 kg and having wide limits of agreement (±4 kg), suggesting significant variability between the devices. Total Body Water and Muscle Mass also exhibit moderate variability, with biases within ±1 kg, but wider limits (±3–±4 kg), while bone mass shows the least variability with narrow limits (±0.5 kg).

Table 6 presents a comparative analysis of body composition parameters between Obese and Normal groups, measured using PBCA and TBCA devices. For the Weight parameter, the Obese group shows a mean of 43.5kg with a high accuracy of 98.23%, while the Normal group has a mean of 29.5 kg with an accuracy of 96.84% (calculated using Equation (14)). The BMI values are 23.9 kg/m^2^ for the Obese group and 15.6 kg/m^2^ for the Normal group, with accuracies of 96.47% and 95.90%, respectively, indicating reliable measurements. BFat% is higher in the Obese group at 30.3%, compared to 21.6% in the Normal group, with accuracies around 95%. FFM shows a slight difference between groups, with the Obese group at 27.47 kg and the Normal group at 22.43 kg, maintaining high accuracy above 95%. The statistical significance (p-values) for all parameters indicates that the differences between PBCA and TBCA measurements are consistent and significant. Overall, the data suggest that PBCA provides reliable measurements across most parameters, with TBW and BMI being particularly effective at distinguishing between Obese and Normal individuals. Despite these differences, the overall mean accuracy percentage of 94.77% suggests a high level of agreement between the devices, which is obtained using Equation (15). The mean average error of 0.884 indicates the average absolute difference in measurements across all parameters determined using Equation (16).

**Figure 8:**
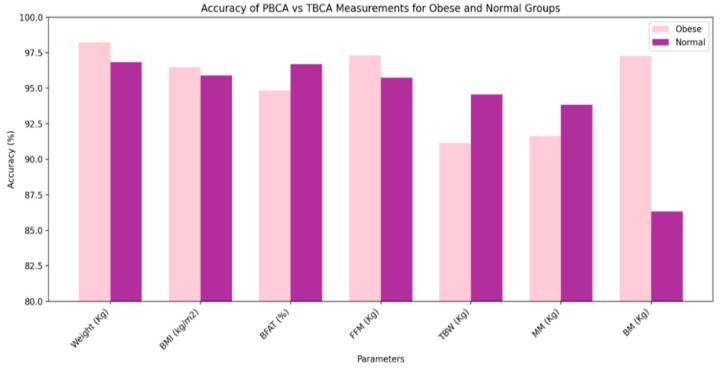
Accuracy comparison of PBCA vs. TBCA measurements for body composition parameters in Obese and Normal groups.

The higher weight in the Obese group is expected due to increased body fat and muscle mass. BMI is a function of weight and height, and the higher BMI in the Obese group reflects increased body mass relative to height. The higher BFat% in the Obese group is consistent with increased adiposity. FFM includes muscle, bone, and water. The Obese group has higher FFM due to the increased muscle mass needed to support greater body weight. TBW is often higher in individuals with more muscle mass as muscle tissue contains more water than fat tissue. The Obese group has higher muscle mass to support greater body weight. Bone mass differences are generally smaller but can be influenced by overall body size and the weight-bearing activities of the subjects. The overall accuracy of the body composition measurements provides valuable insights into the reliability and effectiveness of the PBCA and TBCA devices. The accuracy values, which are consistently high across most parameters, indicate a strong agreement between the two measurement methods. For instance, the accuracy for Weight in the Obese group is 98.23%, and for the Normal group it is 96.84%, reflecting the devices’ ability to produce consistent results. Similarly, BMI accuracy is 96.47% for the Obese group and 95.90% for the normal group, further supporting the reliability of these measurements. These high accuracy values suggest that the PBCA device is a dependable tool for assessing body composition, closely aligning with the traditional TBCA measurements. Figure 8 depicts the accuracy of PBCA vs. TBCA measurements for various body composition parameters in the Obese and normal groups, highlighting differences in measurement consistency across parameters such as Weight, BMI, BFAT, FFM, TBW, MM, and BM.

## 4. Discussion

We analysed body composition in 200 school children, aged 6 to 12, from Tamil Nadu, India, focusing on BMI, FFM, TBW, BFAT%, MM, and BM using a newly developed portable BCA prototype. The children were categorized based on BMI according to CDC guidelines. We validated our prototype against the Tanita MC 980 MA and developed regression equations for each body composition parameter. This study addresses a gap in research on designing prototypes for estimating body composition in children and measuring BCA parameters. Our extensive validation process against the Tanita MC 980 MA BCA, achieving a mean accuracy of 94.77%, demonstrates a high level of precision for a portable device in this demographic, which is a significant contribution to the field. A key innovation of our work is the development and validation of regression equations specifically tailored for estimating body composition parameters in children using our integrated device. Our research offers a holistic approach to pediatric body composition analysis, simultaneously measuring multiple parameters (BMI, Body Fat %, Total Body Water, Fat-Free Mass, Muscle Mass, and Bone Mass) in a single, efficient assessment. This comprehensive evaluation in a portable format represents a novel approach in pediatric health monitoring. This innovation has potential implications for improving accessibility to comprehensive health evaluations in various pediatric settings, from schools to clinics.

Wu et al. conducted considerable research with 554 healthy Asian people ranging in age from 16 to 75 years old. The major goal of this study was to develop an effective regression equation to estimate FFM in a healthy Taiwanese population. A multivariate technique was used to build the FFM prediction model, which is written as FFM = 13.055 + 0.204 * weight + 0.394 * (height2/impedance) − 0.136 * age (with gender included, where female is represented as 0 and male is represented as 1) [38]. Likewise, in this study we found a regression equation for estimating body composition in children. The regression equation of FFM established in this current study for children was FFM = 0.144 (TBW) + 0.413 (MM) + 3.586 (BM) + 0.096 (BMI) + 0.310 (BFat) + 1.166.

An extensive study was carried out by Brunani et al. (2021) encompassing 8303 obese individuals ranging in age from 18 to 90 years. The research investigated resistance (Rz) and reactance (Xc) measures in connection with a variety of characteristics such as gender, age, and BMI classes. Furthermore, the study indicated women had a greater Fat Mass Index (FMI), whereas males had a higher Fat-Free Mass Index (FFMI). FMI and FFMI were also shown to have significant relationships with BMI [39]. Similarly, in this current study, all the body composition parameters, namely, FFM, TBW, BFAT%, MM, and BM were shown to have a significant relationship with the parameter BMI.

The goal of the study conducted by Chen et al. (2017) was to develop a novel BIA model for determining body composition in young, healthy Chinese people. They used isotope dilution, MRI, and DEXA as reference methods for performing this study. The study involved 30 healthy subjects who underwent BIA body composition analysis. The results of the BIA measurements were compared to the results of the three reference methods. To integrate data from all three reference techniques into a single formula, a comprehensive model was developed. The model’s validity and accuracy were evaluated with a larger sample of 209 individuals. The authors developed a regression equation of BIA (Y = 1.072 * X * e^(−k * 1.786/(1.77)^2) + 0.3194 * K^2 − 2.073 * K + 3.713, where X indicated the impedance index, whereas Y reflected total body water (k = 1), fat mass (k = 2), and bone mass (k = 3), which had a prediction accuracy of 93.3% [40]. Riyadi et al. in 2017 conducted a study using a BIA technique for measuring body composition in 10 subjects. The experimental findings show notable repeatability for consecutive measurements, with a standard deviation for fat mass of less than or equal to 0.25%. Furthermore, when the findings of this BIA tool are compared to the results of a hand-to-hand node system, the average absolute difference in fat mass for the total subject group is 0.48%, with the largest absolute differences being 1.22%. When the relative error is standardized to Omron’s HBF-306 as a reference tool, it is less than 2% [41]. Similarly, we also developed a BCA for children using the BIA technique and assessed crucial parameters from it. Moreover, for each body composition parameter, a regression equation was formed. Then, the prototype BCA parameter was validated using Tanita BC 980 MA which also works on the principle of BIA, and the overall accuracy percentage was found to be 94.77%. Moreover, the mean average error of 0.884 indicates the average absolute difference in measurements across all parameters determined for obese and normal children.

This study was limited to 100 individuals in each group (Obese and Normal), which may not be representative of the broader population. Differences in calibration between PBCA and TBCA devices could affect the accuracy of measurements. Variability in measurements due to factors such as hydration status, time of day, and recent physical activity could influence results. Future studies include a more diverse sample in terms of age, gender, ethnicity, and health status to enhance the generalizability of findings. Conducting longitudinal studies to track changes in body composition over time or in response to interventions could provide valuable insights into the dynamics of body composition. In this study, we focused specifically on children classified as either normal weight or obese, excluding those categorized as overweight to streamline the validation process of the portable body composition analyzer (BCA). Future studies can expand on this work by incorporating overweight participants to explore the device’s efficacy across a broader spectrum of body composition categories in children. Employing advanced statistical or machine learning techniques could help identify patterns or predictors of body composition changes that are not apparent with traditional methods.

## 5. Conclusions

A wide variety of body composition parameters of children were measured precisely, employing the developed BCA prototype. The device assessed key indicators such as body mass index, body fat percentage, total body water, fat-free mass, muscle mass, and bone mass. Results from the study demonstrated the prototype’s exceptional performance, achieving a remarkable mean accuracy of 94.77% across all measured parameters. This high level of precision underscores the device’s capability to effectively quantify and evaluate multiple aspects of body composition in young subjects. To validate the prototype’s reliability, the researchers conducted a comparative analysis with the Tanita MC 980 MA BCA, a well-established and widely used body composition analyzer. The comparison revealed a mean average error of approximately 0.884 for measurements taken from both obese and healthy children. This low standard error indicates strong agreement between the prototype and the Tanita BCA, reinforcing the consistency and dependability of the new device’s assessments across different body types. In addition, we developed and validated regression equations for predicting body composition parameters based on related variables, enhancing the analytical capabilities of the device. For instance, the regression equation for BMI (R^2^ = 0.909) incorporates key predictors such as BF% (coefficient = 0.417), FFM (0.358), and BM (7.654), while the BF% prediction model (R^2^ = 0.895) includes FFM (0.560), TBW (0.450), and BMI (0.477). These regression models allow for accurate estimates of body composition, providing flexibility for diverse pediatric health applications. This study highlights the BCA prototype’s potential as a reliable tool for assessing and monitoring children’s body composition. Its high overall accuracy and low standard error, when compared to a recognized industry standard, make it particularly valuable for applications involving diverse populations, including both obese and healthy children. These findings suggest that the prototype could become a significant asset in the field of pediatric body composition analysis, offering researchers and healthcare professionals a dependable instrument for gathering crucial physiological data.

## Figures and Tables

**Table 1:** Category and range established by the Centers for Disease Control and Prevention for Children. Source: Centers for Disease Control and Prevention (CDC). “Defining Childhood Obesity: BMI for Children and Teens”. Available at: https://www.cdc.gov/bmi/child-teen-calculator/bmi-categories.html (20 May 2024) [25].

Category	Range	Boys (n)	Girls (n)	Total (n)
Underweight	Below 5th percentile	5	7	12
Healthy weight	Between 5th–85th percentile	50	50	100
Overweight	Between 85th–95th percentile	9	12	21
Obese	Above 95th percentile	50	50	100

**Table 2:** Anthropometric and body composition characteristics of normal and obese children in the study population (N = 200).

	Total Population (N = 200)			
Para Meters	Obese (n = 100)	>Normal (n = 100)	% Diff.	Sig. P
**Age (years)**	8.441 ± 0.501	8.061 ± 0.011	4.605	0.003
**Weight (Kg)**	43.501 ± 0.707	29.511 ± 3.535	38.322	0.001
**Height (cm)**	126.511 ± 3.355	125.188 ± 2.828	1.051	0.034
**BMI (kg/m^2^)**	23.976 ± 3.392	15.697 ± 1.639	41.736	0.001
**BF (%)**	31.309 ± 3.709	21.644 ± 2.378	37.504	0.001
**FFM (Kg)**	27.473 ± 1.962	22.431 ± 1.084	20.206	0.002
**TBW (Kg)**	18.919 ± 3.976	11.304 ± 1.727	30.392	0.001
**MM (Kg)**	23.255 ± 2.221	15.847 ± 1.577	36.891	0.001
**BM (Kg)**	1.065 ± 0.089	0.745 ± 0.087	35.359	0.002

**Table 3:** Pearson correlation matrix for all obese (n = 100) children of the study.

	Weight	BMI	TBW	FFM	BF%	MM	BM
**Weight**	1.000	-	-	-	-	-	-
**BMI**	0.241 *	1.000	-	-	-	-	-
**TBW**	0.161	0.296 *	1.000	-	-	-	-
**FFM**	0.277 *	−0.047	0.132 *	1.000	-	-	-
**BF%**	0.437 **	0.827 ***	0.319 *	0.438 **	1.000	-	-
**MM**	0.160 *	0.175 *	−0.002	0.527 **	0.444 **	1.000	-
**BM**	−0.007	0.201 *	−0.011	0.166 *	0.382 *	0.030	1.000

*** Correlation significant (*p* < 0.01) level (2-tailed), ** correlation significant (*p* < 0.03) level (2-tailed), * correlation significant (*p* < 0.05) level (2-tailed).

**Table 4:** Regression models for all BCA parameters measured using portable BCA for children.

Parameters	Coefficient	Standard Error	t Stat	*p*-Value	Multiple R	R Square	F Score	Sig. F
**BMI as a dependent variable (N = 200)**								
**Intercept**	−0.833	2.894	−0.288	0.774	0.842	0.909	45.71	0.002
**BF**	0.417	0.086	4.834	0.000				
**FFM**	0.358	0.196	1.827	0.071				
**TBW**	−0.090	0.201	−0.446	0.657				
**MM**	−0.139	0.124	−1.120	0.266				
**BM**	7.654	2.986	2.563	0.012				
**BF% as a dependent variable (N = 200)**								
**Intercept**	−3.081	3.082	−1.000	0.320	0.869	0.895	58.07	0.001
**FFM**	0.560	0.206	2.720	0.008				
**TBW**	0.450	0.210	2.140	0.035				
**MM**	−0.116	0.133	−0.872	0.386				
**BM**	−0.390	3.305	−0.118	0.906				
**BMI**	0.477	0.099	4.834	0.000				
**FFM as a dependent variable (N = 200)**								
**Intercept**	1.166	1.491	0.782	0.436	0.942	0.888	149.35	0.002
**TBW**	0.144	0.103	1.404	0.164				
**MM**	0.413	0.048	8.562	0.000				
**BM**	3.586	1.552	2.310	0.023				
**BMI**	0.096	0.052	1.827	0.071				
**BF**	0.130	0.048	2.720	0.008				
**TBW as a dependent variable (N = 200)**								
**Intercept**	12.052	0.813	14.819	0.000	0.889	0.891	70.97	0.001
**MM**	0.131	0.062	2.104	0.038				
**BM**	6.467	1.437	4.499	0.000				
**BMI**	−0.024	0.053	−0.446	0.657				
**BF**	0.103	0.048	2.140	0.035				
**FFM**	0.142	0.101	1.404	0.164				
**MM as a dependent variable (N = 200)**								
**Intercept**	−9.155	2.204	−4.153	0.000	0.893	0.897	73.75	0.003
**BM**	−1.502	2.554	−0.588	0.558				
**BMI**	−0.095	0.085	−1.120	0.266				
**BF**	−0.069	0.080	−0.872	0.386				
**FFM**	1.061	0.124	8.562	0.000				
**TBW**	0.342	0.163	2.104	0.038				
**BM as a dependent variable (N = 200)**								
**Intercept**	−0.194	0.095	−2.053	0.043	0.874	0.864	60.916	0.001
**BMI**	0.009	0.003	2.563	0.012				
**BF**	0.000	0.003	−0.118	0.906				
**FFM**	0.015	0.006	2.310	0.023				
**TBW**	0.027	0.006	4.499	0.000				
**MM**	−0.002	0.004	−0.588	0.558				

**Table 5:** Absolute, relative, and inter-rater reliability test of prototype BCA (PBCA) and Tanita BCA (TBCA) obtained for obese subjects between two raters for all the seven parameters.

Variable	% Mean Difference (95% CI)	*p*-Value	SEM	MD	Relative Reliability (95% CI)	Inter-Rater Reliability (95% CI)
Weight (PBCA)	3.49 (2.96–4.01)	0.12	0.38	1.05	1.78 (0.78–2.78)	0.95 (0.93–0.96)
Weight (TBCA)	3.97 (3.37–4.57)	0.05	0.49	1.35	2.21 (1.21–3.21)	0.95 (0.93–0.96)
BMI (PBCA)	8.12 (7.47–8.76)	0.06	0.47	1.29	4.11 (3.11–5.11)	0.94 (0.93–0.96)
BMI (TBCA)	6.24 (5.30–7.18)	0.05	0.42	1.18	3.60 (2.60–4.60)	0.94 (0.93–0.96)
BFAT (PBCA)	0.07 (−0.06–0.12)	0.88	0.05	0.13	0.31 (−0.69–1.31)	0.96 (0.93–0.96)
BFAT (TBCA)	0.25 (0.00–0.49)	0.59	0.18	0.49	1.12 (0.12–2.12)	0.96 (0.93–0.96)
FFM (PBCA)	6.74 (6.11–7.36)	0.07	0.45	1.25	3.40 (2.40–4.40)	0.95 (0.93–0.96)
FFM (TBCA)	0.02 (−0.04–0.09)	0.65	0.05	0.13	0.35 (−0.65–1.35)	0.96 (0.93–0.96)
TBW (PBCA)	3.07 (2.79–3.35)	0.22	0.2	0.56	2.15 (1.15–3.15)	0.95 (0.93–0.96)
TBW (TBCA)	−1.93 (−2.07–−1.79)	0.24	0.1	0.28	1.05 (0.05–2.05)	0.96 (0.93–0.96)
MM (PBCA)	2.77 (2.35–3.19)	0.05	0.21	0.58	1.81 (0.81–2.81)	0.95 (0.93–0.96)
MM (TBCA)	−4.38 (−4.75–−4.00)	0.23	0.27	0.75	2.33 (1.33–3.33)	0.95 (0.93–0.96)
BM (PBCA)	3.49 (2.97–4.01)	0.05	0.01	0.03	1.95 (0.95–2.95)	0.95 (0.93–0.96)
BM (TBCA)	1.16 (1.15–1.17)	0.69	0.01	0.02	1.39 (0.39–2.39)	0.96 (0.93–0.96)

**Table 6:** Validation of the proposed portable BCA (PBCA) with the Tanita MC 980 MA (TBCA) for the study subject (N = 200).

Parameters	Obese (n = 100)				Normal (n = 100)				Accuracy (%)	
	PBCA	TBCA	Standard Error	Sig. P	PBCA	TBCA	Standard Error	Sig. P	Obese	Normal
Weight (Kg)	43.501 ± 0.7	42.746 ± 0.3	0.081	0.001	29.511 ± 3.5	30.473 ± 2.8	0.454	0.03	98.23	96.84
BMI (kg/m^2^)	23.976 ± 3.3	24.854 ± 1.6	0.376	0.020	15.697 ± 1.6	16.368 ± 0.6	0.177	0.001	96.47	95.90
BFAT (%)	30.309 ± 3.7	31.959 ± 2.5	0.453	0.003	21.644 ± 2.3	20.954 ± 2.9	0.381	0.039	94.84	96.71
FFM (Kg)	27.473 ± 1.9	26.753 ± 0.6	0.207	0.006	22.431 ± 1.08	23.427 ± 2.1	0.243	0.001	97.31	95.75
TBW (Kg)	18.919 ± 3.9	17.379 ± 4.1	0.575	0.008	11.304 ± 1.7	11.954 ± 2.6	0.318	0.042	91.14	94.56
MM (Kg)	23.255 ± 2.2	21.461 ± 2.9	0.371	0.001	15.847 ± 1.5	14.925 ± 1.9	0.254	0.004	91.64	93.82
BM (Kg)	1.065 ± 0.08	1.095 ± 0.04	0.011	0.002	0.745 ± 0.08	0.863 ± 0.03	0.010	0.001	97.26	86.33

## Data Availability

The data presented in this study are available on request from the corresponding author.
